# Quality of care for major depression and its determinants: a multilevel analysis

**DOI:** 10.1186/1471-244X-12-142

**Published:** 2012-09-17

**Authors:** Arnaud Duhoux, Louise Fournier, Lise Gauvin, Pasquale Roberge

**Affiliations:** 1 CRCHUM (Centre de recherche du Centre Hospitalier de l’Université de Montréal), Edouard-Asselin Pavilion, 264, René-Lévesque Blvd. East, Montreal, QC, Canada H2X 1P1; 2 Université de Montréal, C.P. 6128, succursale Centre-ville, H3C 3 J7, Montreal, QC, Canada; 3 Institut National de Santé Publique du Québec, 190 Crémazie Blvd. East, H2P 1E2, Montreal, QC, Canada; 4 Université de Sherbrooke, 3001, 12e Avenue Nord, J1H 5 N4, Sherbrooke, QC, Canada

**Keywords:** Quality of care, Quality indicator, Major depressive episode, Adequacy of treatment, Multilevel analysis

## Abstract

**Background:**

Numerous studies highlight an important gap in the quality of care for depression in primary care. However, basic indicators were often used. Few of these studies examined factors associated with receiving adequate treatment, particularly with a simultaneous consideration of individual and organizational characteristics. The purpose of this study was to estimate the proportion of primary care patients with a major depressive episode (MDE) who receive adequate treatment and to examine the individual and organizational (i.e., clinic-level) characteristics associated with the receipt of at least one minimally adequate treatment for depression.

**Methods:**

The sample used for this study included 915 adults consulting a general practitioner (GP), regardless of the motive of consultation, meeting DSM-IV criteria for MDE during the 12 months preceding the survey (T1), and nested within 65 primary care clinics. Data reported in this study were obtained from the “Dialogue” project. Adherence rates for 27 quality indicators selected to cover the most important components of depression treatment were estimated. Multilevel analyses were conducted.

**Results:**

Adherence to guidelines was high (>75%) for one third of the quality indicators that were measured but was low (<60%) for nearly half of the measures. Just over half of the sample (52.2%) received at least one minimally adequate treatment for depression. At the individual level, determinants of receipt of minimally adequate care included age, having a family physician, a supplementary insurance coverage, a comorbid anxiety disorder and the severity of depression. At the clinic level, determinants included the availability of psychotherapy on-site, the use of treatment algorithms, and the mode of remuneration.

**Conclusions:**

Our findings suggest that interventions are needed to increase the extent to which primary mental health care conforms to evidence-based recommendations. These interventions should target specific populations (i.e. the younger adults and the elderly), enhance accessibility to psychotherapy and to a regular family physician, and support primary care physicians in their clinical practice with patients suffering from depression in different ways such as developing knowledge to treat depression and adapting mode of remuneration.

## Background

Major depressive episode (MDE) is a very common disorder with a lifetime prevalence estimated at 12.2% [1]. MDE is the leading cause of disease burden in developed nations in terms of years lived with disability [2]. Individuals with depression also commonly experience multiple episodes of relapse and recurrence leading depression to be viewed as a chronic condition [3].

The critical role of primary care in the detection and treatment of depression is now widely recognized [4]. Unfortunately, numerous studies highlight an important gap in depression treatment in primary care settings, where this disorder is often not treated or not adequately treated [5-7]. However, other studies suggest that when primary care for depression is minimally consistent with clinical practice guidelines patients experience fewer symptoms [8-11], improved quality of life [8,9] and functioning [12], and have a reduced risk of relapse or recurrence [13,14]. Guideline consistent primary care is also associated with increased treatment cost-effectiveness [15].

Since the 1990s, a number of countries have developed evidence-based clinical practice guidelines in an effort to improve the quality of care for depression [16-21]. These guidelines have been developed on the basis of clinical research syntheses and expert consensus reports. Optimal strategies for treating depression have been described, in both psychiatric and primary care settings. The two main recognized treatment options for depression are pharmacotherapy and psychotherapy [19].

Clinical practice guidelines are also increasingly used to establish standards for treatment quality at a population level. Quality indicators measure the gap between actual care received by patients and established standards. They provide tools for evaluating processes of care (technical and interpersonal aspects of care delivered to patients) [22,23] according to Donabedian’s triad of structure, process, and outcome to conceptualize the quality of health care [24]. Many authors have underscored the importance of using process indicators for measuring the quality of care [22,23,25-27]. Measurement of quality at the population level cannot address the specificities of each individual or situational peculiarities in which deviating from practice guidelines might be appropriate. Quality indicators must therefore be considered minimal standards of care.

Given the increased interest in the quality of care for mental health problems in the previous decade, there has been a proliferation of measurement indicators [28]. However, no consensus has emerged regarding the most appropriate indicators of quality for the treatment of depression in primary care and few indicators have been subjected to validation efforts [7,28].

One of the main findings of a systematic review of literature on quality indicators for treatment of depression in primary care that we published recently [28] was that most of the studies reviewed used only rudimentary indicators to measure the quality of treatment for depression. This gap was particularly evident in studies assessing the quality of psychotherapy. For example, the majority of studies used a minimum number of visits as an indicator for psychotherapy quality without details on the duration of visits or the type of psychotherapy used. We also noted that the vast majority of studies on quality of treatment for depression did evaluate the quality of pharmacotherapy. However, the accuracy of indicators was uneven. Some details such as number of prescriptions, dosage of antidepressants (ATD), and number of follow-up visits were not always assessed. One consequence of relying on quality measures that are too basic is that important aspects of depression care are left unassessed, providing an incomplete picture of the care depressed individuals receive. In this review, we recommended the development and use of more sophisticated indicators of quality.

Quality indicators that combine elements of both pharmacotherapy and psychotherapy encompass both of the main treatment options and provide a potentially more accurate assessment of depression treatment quality. A number of studies have used indicators that account for the quality of pharmacotherapy and/or the quality of psychotherapy by creating a global quality indicator that measures minimal treatment adequacy [28]. The prevalence rates ranged from 14% to 56% suggesting that a large proportion of people suffering from depression do not receive minimally adequate treatment. Studies including indicators related to patient education about depression also showed large disparities with adequacy rates ranging from 23% to 100%.

Few of these studies examined the various factors associated with receiving adequate treatment, and the results obtained in this regard were conflicting. Andersen’s Behavioral Model of Health Care [29] is a well-known model, developed for utilization of care studies. It has been used in some studies related to adequacy of treatment for depression to identify individual factors potentially associated with adequate treatment [7,30-32]. The model considers an individual’s use of services as a function of their predisposing characteristics, enabling characteristics, and need for care. Even if multilevel analysis is particularly well suited to investigate both patient- and clinic-level factors influencing quality of depression treatment in primary care, few studies used this analysis method in this context. Furthermore, they considered only clinician burden at the clinic level [33] or did not focus on depressive disorder only [32,34].

The objectives of this study are therefore:

to estimate the proportion of primary care patients meeting DSM-IV criteria for major depressive disorder who receive adequate treatment as assessed by quality indicators derived from clinical practice guidelines

 to examine the individual and organizational (i.e., clinic-level) characteristics associated with the receipt of at least one minimally adequate treatment for depression.

## Methods

### Design

Data reported in this study were obtained from the “Dialogue” project [35], a research program consisting of three main interrelated components: 1) a contextual study consisting of a qualitative examination of primary mental health care services and contextual factors perceived to influence the implementation of a provincial mental health reform in 15 local service networks (LSN) of the province of Québec (the LSN were selected for their diversity of contexts: urban to remote, population size, specialized resources availability, etc.); 2) a cross-sectional organizational survey conducted in 76 primary care clinics located in the 15 LSN to describe the variability in organizational models of primary care; and 3) a client survey that examined the experience of care and evolving mental health status among a cohort of adults with anxiety and depressive disorders. Patients were recruited in 67 of the 76 clinics that had participated in the organizational survey and who accepted the recruitment of participants in their waiting room. Following incept into the cohort (T0), the tracking process involved three telephone/web interviews conducted at six-month intervals (T1, T2, and T3).

Data for the current investigation were drawn from the organizational survey and from the waiting room interview (i.e., inception into the cohort, T0) and the first telephone/web interview (T1) of the client survey. The procedures were approved by the Human Research Ethics Committees of all regional authorities involved in the project (Agence de santé de des services sociaux de Montréal; Centres de santé de des services sociaux de Chicoutimi, Sherbrooke, Gatineau, Laval, Saint Jérome, Jeanne-Mance, Lac-Saint-Jean-Est, Pointe-De-L’ile, Bordeaux-Cartierville-Saint-Laurent, Ste Therese-De-Blainville, Pierre Boucher, Haut-Richelieu-Rouville, Baie des Chaleurs, La Pommeraie; Hospital Notre-Dame and Hospital Sacré-Coeur). Study participants provided written informed consent. These considerations are in keeping with the ethical principles set out in the Declaration of Helsinki [36].

### Participants

#### Sampling of clinics

For the organizational survey, recruitment letters and questionnaires were sent to the 285 primary care clinics of the 15 selected LSN. In 67 clinics, the respondent most knowledgeable about the clinic’s organization and functioning completed a standardized questionnaire and allowed for the recruitment of participants in the waiting room. The organizational questionnaire was adapted from a previous study [37] to primary mental health care. It consisted of fifty questions divided into five sections: resources and organizational structure, services and practices, interorganisational collaboration, vision / values and location of the clinic. The information was collected from November 2007 to June 2008.

#### Sampling of persons within clinics

**Figure 1:**
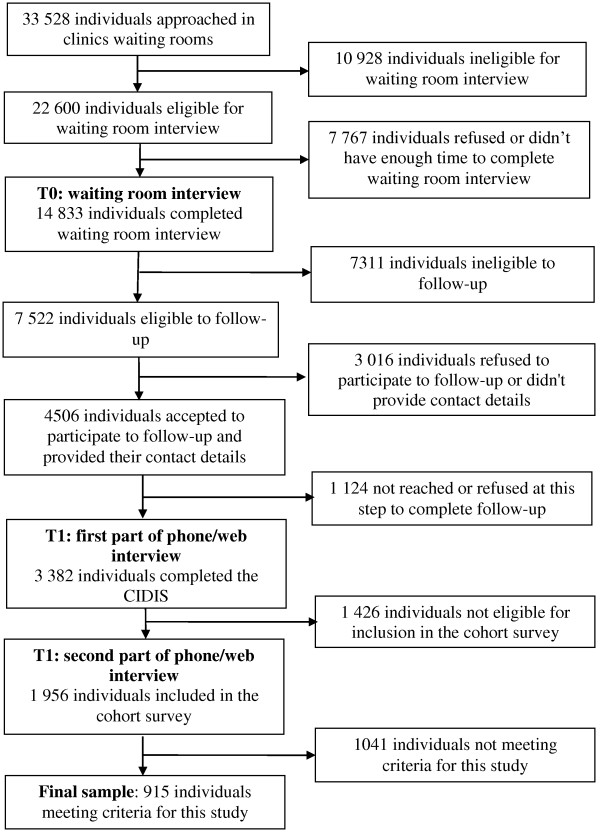
Recruitment flow-chart, Dialogue Project.

Participants were recruited between March and August 2008 in the waiting rooms of the 67 primary care medical clinics (T0) during randomly chosen periods to ensure proper representation of every day of the week and of different period of the day (morning, afternoon, and evening). The recruitment flowchart appears in Figure1.

##### Eligibility for the waiting room interview (T0)

French and English speaking adults (18 years and over) seeking care for themselves from a general practitioner (GP), regardless of the motive of consultation, were approached by trained research assistants to complete a brief self-administered questionnaire. From the 22 600 eligible patients approached, 67.4% (n = 14 833) completed the questionnaire.

##### Eligibility for follow-up (first part of T1)

Patients were invited to participate in the first part of T1 (n = 7 522) if their usual source of care was one of the participating clinics and if they met at least one of the following characteristics: i) high level of depressive or anxiety symptoms in the past week according to the Hospital Anxiety and Depression Scale (HADS) [38] (The HADS consist of two sub-scales; seven items measure anxiety symptoms (HADS-A) and seven items measure depression symptoms (HADS-D) in the past week. Each item is scored on a four points scale (0 to 3), with the total score ranging from 0 to 21 for each sub-scale. A higher score indicates major distress and a higher probability to present an anxiety or depressive disorder. An individual with a score of 8 or more on a sub-scale is considered to have a possible disorder. The scale performs well to evaluate the symptom severity of anxiety disorders and depression in various community settings and primary care [39] and presents good internal consistency, reliability and convergeant/discriminant validity [40]); ii) taking medication for depressive or anxiety problems within the year previous to the survey; iii) having been diagnosed with a depressive or anxiety disorder by a physician; iv) consulting a health professional (GP, psychiatrist, psychologist, etc.) for mental health reasons within the year prior to the survey.

Among them, 4 506 (59.9%) accepted to participate to the follow-up and provided their contact details in the waiting room questionnaire. After 2–4 weeks, we were able to contact by telephone and/or email 3 382 (75.1%) individuals for a first interview (T1). A total of 2 396 (70.8%) telephone interviews and 986 (29.1%) web questionnaires were completed. The first part of the structured interview was used to determine whether respondents had a high probability of meeting DSM-IV criteria for an anxiety or depressive disorder.

##### Eligibility for inclusion in the cohort (second part of T1)

The interview then continued with the 1 956 people meeting any of the following criteria: i) presence of at least one of the diagnoses assessed in the last 12 months (MDE, generalized anxiety disorder (GAD), agoraphobia, social phobia (SP) and panic disorder (PD). The CIDIS (Composite International Diagnostic Interview Simplified) [41] was used to identify psychiatric disorders according to the DSM-IV classification [42]); ii) a high level of anxiety or depression symptoms combined with medication, diagnosis by a health care professional, or DSM-IV criteria for anxiety or depression in the past 24 months. Data collected pertained to patient health (symptoms, disabilities, functioning, length of episode, comorbidity), mental health care trajectory (number and types of professionals seen, number of visits, referrals from one professional to another, medication), accessibility of care and continuity and responsiveness of services.

##### Sample used for this study

For the present study, the final sample included 915 adults consulting a GP, regardless of the motive of consultation, meeting the criteria for MDE during the 12 months preceding the survey (T1), and nested within 65 primary care clinics (Two clinics were excluded because we recruited only participants with an anxiety disorder and none with MDE).

### Measures

#### Quality indicators for depression treatment

Quality indicators for depression treatment were established from Canadian clinical practice guidelines [3,19] and previous studies [7,28]. Those quality indicators were selected to cover the most important components of depression treatment and pertain to detection of depression; ATD medication prescribed, including its dosage and follow-up; psychotherapy; information/education received by the patient; the consideration of patient’s preferences and the receipt of advice or encouragement to do physical exercise to improve well-being, emotions and mental health. Rates of adherence to 27 indicators were evaluated with patients’ self-reported data. Each indicator was considered only regarding the specific patient population to which a care process applied. Their description appears in Table1. 

**Table 1:** Quality of treatment for depression according to different indicators based on patients’ self-reported data in the Dialogue Project in 2008

**Quality indicator**	**Patients (n)**	**Observed% (n)**	**Indicator description**
Detection of depression	915	68.1% (623)	In the past 12 months, the respondent was told s/he was suffering from depression OR received an antidepressant prescription
**Use of services**			
Use of services for mental health reason	915	86.2% (789)	At least one consultation for mental health reason in the past 12 months (family doctor/ general practitioner, psychiatrist, other physicians, psychologist, nurse, social worker/counselor/ psychotherapist, other health provider or professional)
Watchful waiting (monitoring of untreated patients)	137	40.1% (55)	3 or more medical consultation for mental health reason in the past year (among untreated respondents, i.e. respondents with at least one consultation for mental health reason in the past 12 months but no antidepressant prescription and no help in the form of psychotherapy or counseling)
**Psychotherapy**			
Any form of psychotherapy or counseling	789	56.1% (443)	Help in the form of psychotherapy or counseling in the past 12 months (among those with at least one consultation for mental health reason)
Adequate length session for psychotherapy or counseling	443	88.9% (394)	At least one session lasting 15 minutes or more of psychotherapy or counseling with one or other of the professionals consulted (among those who received help in the form of psychotherapy or counseling)
At least one of the recommended psychotherapy	394	83.2% (328)	Cognitive behavior therapy AND/OR Interpersonal therapy (among those who received at least one session for counseling of adequate length)
Complete course of psychotherapy	443	60.7% (269)	12 or more consultations for mental health reason in the past year. According to the Canadian recommendations, a minimum of 12 visits is required for a full course of psychotherapy (among those who received help in the form of psychotherapy or counseling)
Adequate psychotherapy	443	49% (217)	At least one of the recommended psychotherapy + complete course of psychotherapy (among those who received help in the form of psychotherapy or counseling)
**Medication**			
Antidepressant prescription in the past year	915	59.5% (544)	In the past 12 months, the respondent received an antidepressant prescription
Adequate follow-up of the prescription	544	77.9% (424)	The respondent consulted 3 times or more the professional who prescribed the medication (among those who received an anti-depressant prescription)
Compliance support	544	80.1% (436)	Any one or other of the professionals who prescribed the medication helped the respondent follow the course of treatment (among those who received an anti-depressant prescription)
Patient education about antidepressant prescription			Any one or other of the professionals who prescribed the medication provided information on the subject of… (among those who received an anti-depressant prescription)
The effectiveness of treatment	544	72.8% (396)	
The possible side effects	544	74.8% (407)	
The probable length of treatment	544	59.2% (322)	
Side effects you may expect to experience if you stop taking the medication of your own accord	544	61.6% (335)	
Adequate length of treatment	83	59% (49)	The respondent have been taking the antidepressant medication for 180 days or more (among those who stopped their treatment)
Adequate dosage of antidepressant medication	458	88.9% (407)	The respondent received at least one antidepressant prescription at the minimum recommended dosage (among those taking antidepressant medication at the time of interview)
**At least one minimally adequate treatment**	789	60.5% (477)	Adequate psychotherapy AND / OR Antidepressant prescription in the past year with adequate follow-up (≥ 3 times) (among those with at least one consultation for mental health reason or among the entire sample)
	915	52.2% (477)	
**Patient education - information**			
General patient education -information	789	62.5% (493)	In the past 12 months, the respondent received information about mental health problems, existing treatments or available services (among those with at least one consultation for mental health reason)
Specific patient education - information			The respondent received information about … (among those who received general education – information)
Anxiety	493	72.8% (359)	
Depression	493	83.6% (412)	
Medication	493	78.7% (388)	
Psychotherapy	493	55.6% (274)	
Support and self-help groups in your area	493	39.8% (196)	
Information sources such as books and Internet sites	493	50.3% (248)	
Consideration of patient’s preferences	493	47.1% (232)	The respondent received information about medication AND psychotherapy
Advice or encouragement to do physical exercise	789	74.7% (589)	With any one of the professionals, the respondent received advice or encouragement to do physical exercise to improve well-being, emotions and mental health (among those with at least one consultation for mental health reason)

#### Dependent variable

Receiving at least one minimally adequate treatment was the dichotomous dependent variable used for the second objective and was defined as followed: minimally adequate pharmacotherapy (receiving a prescription for an ATD medication in the past 12 months plus at least 3 medical visits) and/or minimally adequate psychotherapy (12 or more consultations for mental health reason in the previous year and at least one of the recommended psychotherapies). The three required visits for those receiving ATD medication is based on the observation that this is the minimum necessary to monitor effectiveness and side-effects. Similarly, according to the Canadian recommendations, a minimum of 12 visits is required for a full course of psychotherapy.

#### Individual-level characteristics

Andersen’s Behavioral Model of Health Care Use [29] was used to identify individual factors potentially associated with adequate treatment. Predisposing factors selected included age (18–24; 25–44; 45–64; 65+ years); sex; educational attainment (High school or less; College; University), and marital status (Married / Living common-law; Widowed / separated / divorced ; Single).

Perception of economic situation (Well off / meet basic needs - poor / very poor), having a family physician and having a supplementary insurance coverage were selected as enabling factors.

Need for care factors included the number of co-morbid chronic illnesses (0; 1; 2; 3 or more); suffering from at least one co-morbid anxiety disorder (GAD, agoraphobia, SP, PD); perceived mental health as poor or fair; presenting a MDE in the previous 6 months, first occurrence of depressive symptoms more than 5 years ago and the severity of depressive symptoms, measured as a continuous variable with the HADS.

#### Organizational (i.e., clinic-level) characteristics

Several types of variables can affect the adequacy of depression treatment at the clinics level (level 2). We classified those variables under three categories: barriers, resources, and practices. Among the barriers to adequacy, the two variables considered were the lack of time for follow-up and the inadequate mode of remuneration to offer an “optimal level” of care for patients suffering from anxiety or depressive disorders. Two dummy variables were created to contrast “Not at all / Slightly” and “Fairly” to “Highly” for multilevel analysis. Among the resources, we examined the presence of a case manager for patients suffering from anxiety or depressive disorders which should increase the quality by ensuring closer follow-up, the availability of psychotherapy on-site and the type of clinic. Finally a variable on practice was considered which should have a more direct influence on the quality: the number of GPs using treatment algorithms with individuals suffering from anxiety or depressive disorders, categorized as “None/Some” – “All/Most”.

### Statistical analyses

Descriptive statistics and rates of adherence to the 27 indicators were computed with PASW (Predictive Analytics SoftWare) Statistics 18.0. Given the hierarchical structure of the dataset, i.e. individuals nested within primary care clinics, multilevel analyses were conducted using HLM (Hierarchical Linear and Nonlinear Modeling) 6.07 software. The model building followed a step-up approach as suggested by Raudenbush and Bryk [43]. The first model (or “empty” model), in which no predictor variables were specified, allowed to explore whether or not there were variations between clinics in likelihood of receipt of minimally adequate treatment for depression. In the second step (model with level-1 factors), after testing for collinearity, individual correlates were examined in the following order: predisposing factors, enabling factors, and need factors. Severity of depressive symptoms, the only continuous variable, was centered around the grand mean. Dummy variables were created for variables with more than 2 categories. Patient-level covariates with p-values < .1 were included in the model. Although not reaching this criterion, sex was included because it is customary to control for this variable in multivariate analysis.

In the same way, clinics characteristics were examined in the third step while adjusting for individual-level correlates (final model with level-1 and level-2 factors). Non-linear Bernoulli analyses for a dichotomous outcome variable were used. Within clinics, samples varied between 1 and 42 respondents. Average within clinics sample comprised 14.1 individuals.

## Results

### Description of the sample of participants and clinics

Baseline characteristics of the 915 patients and 65 clinics are shown in Table2. People meeting the criteria for MDE in the past year were mostly female (75%), married or living common-law (51%) and 45% had completed a high school education. The majority (78%) had at least one chronic medical condition with 35% having three or more. More than half of respondents (55%) also met criteria for an episode of anxiety disorder in the past year. For 70% of the sample, the first symptoms of depression appeared more than 5 years ago. This implies that many of these patients could be characterized as complex cases. These characteristics also confirm that patients were not all recruited at the same stage of their MDE. Indeed, the average score on the HADS-D was less than the cut-point of 8 that would indicate a possible depression and for 25% of the sample, the MDE occurred more than 6 months ago.

**Table 2:** Characteristics of respondents meeting DSM-IV MDE criteria (n = 915) and of clinics where they sought care (n = 65) in the Dialogue Project in 2008

**Characteristics of respondents**	
**Predisposing factors**	
Age (mean (sd))	43.8 (13.9)
Sex	
Female	75%
Male	25%
Education level	
High school or less	45%
College	29%
University	26%
Marital status	
Married / living common-law	51%
Widowed / separated / divorced	22%
Single	28%
**Enabling factors**	
Perception of Economic Situation	
Poor or Very Poor	30%
Well off / meet basic needs	70%
Have a family physician	
Yes	83%
No	17%
Have a supplementary insurance coverage	
Yes	58%
No	42%
**Need factors**	
Severity of depressive symptoms (HADS depression sub-scale (mean (sd))	7.7 (4.4)
At least one comorbid Anxiety Disorder(GAD, Agoraphobia, SP, PD)	
Yes	55%
No	45%
Perceived Mental Health as	
Poor or Moderate	43%
Good or Very Good or Excellent	57%
Depression Episode	
in Previous 6 months	75%
Between 6 and 12 months ago	25%
First Occurrence of Symptoms	
> 5 years	70%
≤ 5 years	30%
Comorbid Chronic Illnesses	
0	22%
1	23%
2	20%
3 or more	35%
**Characteristics of clinics**	
Psychotherapy available on-site	
Yes	62%
No	38%
Presence of a case manager for patients suffering from anxiety or depressive disorders	
Yes	46%
No	54%
Number of GP using treatment algorithms with individuals suffering from anxiety or depressive disorders	
None/Some	68%
All/Most	32%
Inadequate mode of remuneration to offer an “optimal level” of care for patients suffering from anxiety or depressive disorders	
Not at all / Slightly	14%
Fairly	31%
Highly	35%
Lack of time for follow-up to offer an “optimal level” of care for patients suffering from anxiety or depressive disorders	
Not at all / Slightly	14%
Fairly	46%
Highly	40%

The 65 clinics retained for this study included 21 community clinics (public funded clinics characterized by a multidisciplinary and collaborative practice), 14 family medicine groups (groups of physicians who work closely with nurses, in an environment that promotes the practice of family medicine to registered individuals), 9 large private clinics (6 GPs and more), 13 small private clinics (2 to 5 GPs), and 8 “solo” clinics (one GP). Large and small private clinics and solo clinics are characterized by a variable mix of walk-in and family practice in a private context. We found that for a large proportion of the clinics, the inadequate mode of remuneration and the lack of time for follow-up were significant barriers. In 32% of clinics, all or most of the GPs used treatment algorithms for the treatment of MDE or anxiety disorders. For 62% of the clinics, psychotherapy was available on-site and that may be explained by a large proportion of clinics being community clinics or large family medicine groups.

### Prevalence of minimally adequate treatment according to quality indicators

**Figure 2:**
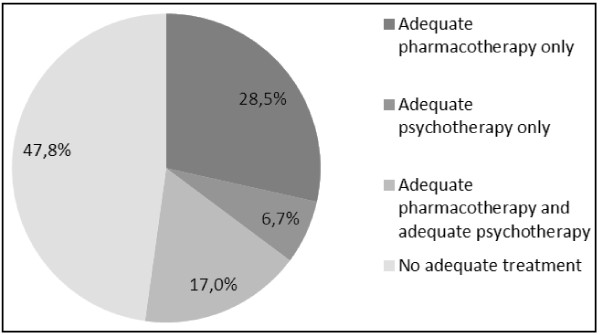
Receipt of minimally adequate treatment for depression among a sample of 915 adults consulting in primary care and meeting criteria for past year MDE in the Dialogue Project in 2008.

In our sample of primary care adults meeting the criteria for MDE in the past 12 months, 68% were detected, 86% had a consultation for mental health reasons, 49% of those treated by psychotherapy received adequate psychotherapy, and 78% of those treated by ATD medication received adequate follow-up (Table1). Overall, 28.5% received minimally adequate pharmacotherapy only, 6.7% received minimally adequate psychotherapy only, and 17% received both (Figure2). More than half of the respondents received some information about mental health problems, existing treatments or available services (62.5%) and almost 75% received advice or encouragement to do physical exercise to improve well-being, emotions, and mental health.

### Empty model – variation across clinics in the likelihood of receiving at least one minimally adequate treatment

Findings showed non-significant between-clinic variation in the likelihood of receiving at least one minimally adequate treatment (level-2 variance component = 0.015; p > 0.5).

The average probability of receiving at least one minimally adequate treatment was 52.1%. Computation of the 95% plausible value range [43] indicated that this probability varied between 46% and 58% across clinics. However, since the variance component is not significantly different from 0, the plausibility value range reflects sampling variance and not differences between the clinics due to a real difference in quality.

### Individual and clinic characteristics associated with the likelihood of receiving at least one minimally adequate treatment

Table3 presents results regarding the association of patient predisposing characteristics, enabling resources, and need factors as well as clinic characteristics and the receipt of at least one minimally adequate treatment.

**Table 3:** Factors associated with minimally adequate treatment for 915 respondents meeting DSM-IV MDE criteria nested in 65 clinics in the Dialogue Project in 2008

	**Model with level 1 factors only**			**Final model**		
	**Coefficient**	**OR**	**95% CI**	**Coefficient**	**OR**	**95% CI**
**Individual characteristics**						
Intercept	−0.72**	0.49	0.31 - 0.78	−1.34***	0.26	0.14 - 0.48
Age						
18-24	−0.43	0.65	0.4 - 1.05	−0.50*	0.61	0.37 - 0.99
25-44 (ref)	-	1.00	-	-	1.00	-
45-64	−0.18	0.83	0.61 - 1.13	−0.20	0.82	0.60 - 1.11
65+	−1.33***	0.27	0.14 - 0.52	−1.45***	0.23	0.12 - 0.47
Sex						
Male	−0.06	0.94	0.68 - 1.3	−0.13	0.88	0.63 - 1.22
Female (ref)	-	1.00	-	-	1.00	-
Has a family physician						
Yes	0.47*	1.61	1.1 - 2.36	0.52**	1.68	1.14 - 2.48
No (ref)	-	1.00	-	-	1.00	-
Has a supplementary insurance coverage						
Yes	0.55***	1.73	1.3 - 2.3	0.53***	1.70	1.27 - 2.27
No (ref)	-	1.00	-	-	1.00	-
At least one comorbid Anxiety Disorder						
Yes	0.58***	1.8	1.35 - 2.39	0.58***	1.79	1.35 - 2.39
No (ref)	-	1.00	-	-	1.00	-
Severity of depressive symptoms (HADS depression sub-scale)	0.07***	1.07	1.04 - 1.11	0.07***	1.07	1.04 - 1.11
**Clinics characteristics**						
Psychotherapy available on-site						
Yes				0.38*	1.46	1.06 - 2.02
No (ref)				-	1.00	-
Number of GP using treatment algorithms with individuals suffering from anxiety or depressive disorders						
All/Most				0.40*	1.49	1.07 - 2.08
None/Some (ref)				-	1.00	-
Inadequate mode of remuneration to offer an “optimal level” of care for patients suffering from anxiety or depressive disorders						
Not at all / Slightly				0.46*	1.58	1.07 - 2.35
Fairly				0.29	1.33	0.91 - 1.95
Highly (ref)				-	1.00	-

* p < 0.05 **p < 0.01 ***p < 0.001.

Predisposing factors included only age: compared to middle age people, younger (OR = 0.61; 95% CI [0.37 - 0.99]) and older people (OR = 0.23; 95% CI [0.12 - 0.47]) were significantly less likely to receive adequate treatment for depression. Among the enabling factors, having a family physician (OR = 1.68; 95% CI [1.14 - 2.48]) and having a supplementary insurance coverage (OR = 1.70; 95% CI [1.27 - 2.27]) were both associated with more adequate treatment, as well as two need factors: severity of depressive symptoms (OR = 1.07; 95% CI [1.04 - 1.11]) and suffering from at least one comorbid anxiety disorder (OR = 1.79; 95% CI [1.35 - 2.39]).

There were no significant differences between the model with only level 1 factors and the full model, indicating that level 2 variable effects are independent from level 1 variables effects. Three clinic characteristics were associated with adequacy of treatment: psychotherapy available on-site (OR = 1.46; 95% CI [1.06 - 2.02]); all or most GPs using treatment algorithms with individuals suffering from anxiety or depressive disorders (OR = 1.49; 95% CI [1.07 - 2.08]); and the mode of remuneration to offer an “optimal level” of care for patients suffering from anxiety or depressive disorders perceived as “not at all” or “slightly” inadequate (OR = 1.58; 95% CI [1.07 - 2.35]).

The presence of a case manager for patients suffering from anxiety or depressive disorders was not associated with the adequacy of treatment as we define it. Neither were the lack of time for follow-up or the type of clinic.

**Figure 3:**
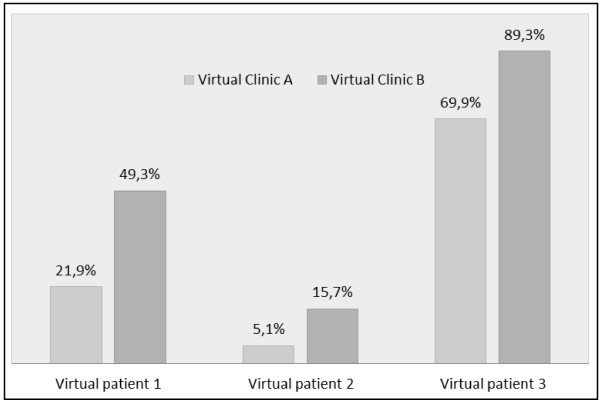
**Estimated probability of receipt of at least one minimally adequate treatment for 3 virtual patients meeting criteria for past year MDE across two virtual clinics in the Dialogue Project in 2008. ***Legend *: **Virtual patient 1 (“*Standard *” *patient *) **: ·Female, Aged between 25 and 44, No family physician, No supplementary insurance coverage, No comorbid anxiety disorder, Grand mean score on the HADS scale. **Virtual patient 2 (“*Low probability*” *Patient*)**: ·Male, Aged 65 or more, No family physician, No supplementary insurance coverage, No comorbid anxiety disorder, Grand mean minus 1 as score on the HADS scale. **Virtual patient 3 (“*High probability*” *Patient*)**: ·Female, Aged between 25 and 44, Family physician, Supplementary insurance coverage, Comorbid anxiety disorder, Score on the HADS scale = 15. **Virtual clinic A (“*Worst*” *clinic*)**: No psychotherapy on-site, None or some GP using treatment algorithms with individuals suffering from anxiety or depressive disorders, Mode of remuneration to offer an “optimal level” of care for patients suffering from anxiety or depressive disorders perceived as highly inadequate. **Virtual clinic B (“*Best*” *clinic*)**: Psychotherapy on-site, All or most of GP using treatment algorithms with individuals suffering from anxiety or depressive disorders, Mode of remuneration to offer an “optimal level” of care for patients suffering from anxiety or depressive disorders perceived as not at all or slightly inadequate.

To illustrate the impact of clinics characteristics, we predicted the likelihood of receiving at least one minimally adequate treatment for three virtual patients as a function of clinic characteristics using estimates from the final multilevel analysis model (Figure3). The first virtual patient is a “standard” patient (i.e. in the reference category for all level-1 variables). The second patient has a low probability of receiving minimally adequate treatment. For the third patient, this probability is high. We contrasted those three patients in two virtual clinics: virtual clinic A which doesn’t have the attributes associated with increased probability of receipt of minimally adequate treatment according to the multilevel model (i.e. in the reference category for all level-2 variables), and virtual clinic B which has those attributes.

The probability of receiving at least one minimally adequate treatment varies between 5.1% and 89.3% depending on the virtual patient and the virtual clinic. Whatever the characteristics of the patient, the increase in probability of receipt of minimally adequate treatment associated with clinic characteristics is substantial. This probability is more than doubled for virtual patient 1 and multiplied by 3 for virtual patient 2, the "low probability" patient. Even patients with a high probability of adequate treatment, as virtual patient 3, can benefit from being treated in clinics with the most favorable characteristics, with a probability of adequate care increased by almost 20%.

## Discussion

The first objective of this study was to estimate the proportion of primary care patients meeting DSM-IV criteria for MDE who receive adequate treatment as assessed by indicators derived from clinical practice guidelines. The rates reported for the 27 indicators, with many of them reported for the first time for patient suffering from depression and consulting in primary care, offer a benchmark for future studies or quality improvement programs.

The second objective was to examine the individual and organizational characteristics associated with the receipt of at least one minimally adequate treatment for depression. Two enabling variables were significantly associated with the outcome indicating that difficulty of access to uncovered care and to a family physician represents a barrier to depression treatment quality. The fact that two need factors are significantly associated with the outcome suggests that people more in need receive more adequate care. Finally, three clinic-level factors were significantly associated with more adequate care, pointing important target to services quality improvement for patient suffering from depression.

### Prevalence of adequate treatment according to quality indicators

Adherence to guidelines was high (>75%) for one third of the quality indicators that were measured but was low (<60%) for nearly half of the measures, pointing to specific targets for quality improvement. For example, untreated patient are monitored (“watchful waiting”) in only 40% of cases, which is lower than previously reported [10].

Educating patients about depression and its treatment is often promoted in practice guidelines and its evaluation seems relevant. Just over 60% of respondents who consulted for mental health reasons reported receiving information about mental health problems, existing treatments or available services. Although this proportion is almost three times higher than previously reported [44,45], there remains room for improvement. In our sample, the proportion of patients who received education regarding pharmacotherapy (on the topic of side effects, the expected efficacy of medication, the consequences of discontinuing treatment prematurely, etc.) ranged between 59% and 75% and were lower than estimates observed in the Netherlands by Smolders et al. [46]. In their study, proportions ranged from 93% to 98%, but physician self-report data were used. Dickinson et al. [33] observed significantly lower proportions for this type of indicator (43%) when using data that were self-reported by patients. This difference in prevalence may demonstrate a difference in perception between the education practitioners think they have provided and that which patients feel they have received.

We didn’t find previous data on advice or encouragement to do physical exercise to improve mental health for this type of population. In our study, even if evidence of the benefits of physical activity for MDE is recent [47-49], we found a surprisingly high rate on this indicator: physical activity was recommended to three quarters of the sample.

Some indicators of pharmacotherapy quality revealed good results. As demonstrated in other studies [50-52], dosage of ATD is often adequate. The follow-up recommended is often described in the guidelines as this aspect of depression care is critical given its role in preventing relapse and recurrence and ensuring patient safety [3,21]. In our sample, we found a high proportion of adequate follow-up after a prescription of ATD in the past year. Even if this result is similar to those found by Hepner et al.[10], indicators related to the intensity of follow-up have often suggested poor performance on this aspect of care [33,53,54]. However, shorter periods (3 or 6 months) were considered in those studies to complete the number of follow-up visit required.

This study is one of the first to provide detailed data on psychotherapy quality [28]. At 56%, our result is similar with that reported by Olfson et al. [55] who showed that 60% of depressed individuals that had had at least one contact with the health system for their depression had received psychotherapy over a period of 12 months. Some studies found lower proportions, ranging from 18% to 28%, but those lower rates can be explained by a period of interest of only 6-months [8,13,56] or the consideration of a larger population of people suffering from depression (not only service users) as denominator [57].

We found a higher proportion of patients receiving the recommended types of psychotherapy among people receiving psychotherapy than Hepner et al. [10] (83% vs 55%), but their indicator considered only cognitive-behavioral therapy whereas we considered also interpersonal therapy as adequate.

The proportion of patients who received at least one adequate treatment among those who received a consultation for mental health reason (60.5%) is in the higher range of earlier findings [28]. When comparing rates of quality indicators for depression treatment, we have to be cautious as we demonstrated earlier [7,28] that there seems to be three main factors that lead to divergent results between studies, namely the populations studied, the indicators used, and the sources of data used. However our results support the conclusion that a significant proportion of persons suffering from depression do not receive treatment considered to be minimally adequate. This finding is all the more alarming considering that it is based on indicators defined using minimal criteria for quality and as such the actual quality of care may be even lower.

### Individual characteristics associated with the likelihood of receiving at least one minimally adequate treatment

Identifying the factors associated with adequate treatment is a key factor for the development of interventions to improve mental health treatment for patients suffering from a MDE.

As in previous studies [57,58], we found that age was associated with treatment adequacy: younger and especially older people were less likely to receive adequate care for depression. However, young adults and elderly are less likely to use health services for mental health reasons [59]. This can explain our finding, as our indicator of minimally adequate treatment relies partly on a minimal number of visits. In a study of primary care patients with anxiety or depression, Prins et al. [60] showed that the individuals in the 18–24 years group are significantly more likely to perceive a need for mental health care, especially for information about mental illness, its treatment and available services compared to the older age groups. The observation that young adults would like to receive more information but are less likely to receive adequate care for depression suggests that this group should be prioritized in efforts to improve the quality of care.

In our multilevel model, two enabling variables were significantly associated with a higher probability of receipt of adequate treatment for depression: having a family physician and having supplementary insurance. The province of Quebec has a universal health insurance for all citizens to obtain medical services and hospital care they need. However, access to a regular family doctor remains a challenge in Quebec and only 73.2% (95% CI [71.1% - 75.2%]) of the population has one [61]. In our sample, this proportion was higher (83%), which can be explained by the recruitment of respondents in the waiting rooms of primary care clinics. However, the results of this study suggest that improving access to family physicians may increase the likelihood of receiving adequate treatment for depression and reinforce the relevance of current efforts in this direction.

The literature suggests that the effect of insurance on quality is inconsistent. Having a private health insurance is a factor for adequacy of care found in several studies [58,62,63]. On the contrary, other authors [7,57] showed that those without private insurance for health care were not less likely to receive adequate treatment for depression. In Quebec, private psychotherapy sessions are not covered by the universal health insurance. The consideration of psychotherapy in our indicator of minimally adequate care may explain why having supplementary insurance is associated with adequate care.

On a positive note, it is reassuring to note that the other predisposing factors (sex, education, and marital status) and perceived poverty are not associated with the receipt of adequate treatment for depression.

The fact that two evaluated need factors, severity of depressive symptoms and comorbid anxiety disorder, were retained in the multilevel model is encouraging: people more in need are more likely to receive adequate care. It is a well-known fact that anxiety and depression are often co-occurring [64]. In our sample, more than half of the respondents also had experienced at least one comorbid anxiety disorder in the last year, which is associated with adequacy of treatment for depression. Other investigators [7,58,63,65] found the same association. Young et al. [58] suggested that depressive symptoms were easier to detect in people presenting both conditions and that those requiring treatment for anxiety may be more willing or have more opportunities to receive treatment for their depressive disorder. In our study, we evaluated need of care with objective clinical factors only. Considering the patient’s perceived needs for care could provide a different perspective, as individuals meeting DSM-IV criteria for MDE do not necessarily want help or believe they need therapy. Indeed, in a similar study performed in the Netherlands, Prins et al. [32] demonstrated that patients’ perceived needs for care were more strongly associated with the delivery of guideline-concordant care for anxiety and depression than clinical need factors.

### Clinic characteristics associated with the likelihood of receiving at least one minimally adequate treatment

Three clinic-level factors were significantly associated with more adequate care, pointing to important targets for quality improvement in services for patient suffering from depression.

Among the resources, psychotherapy on-site is a factor related to adequate care for depression. The important role of evidence-based psychotherapy in the treatment of depression is underscored in many clinical guidelines [3,21]. Richards et al. [66] showed that implementation of evidence-based psychological therapies into routine service settings are associated with improvement of depressive symptoms. However access to those treatments is still limited [67] and poor coordination with mental health specialists can explain less than optimal depression care [45,68]. The ability to deliver psychotherapy on site could improve the accessibility of psychotherapeutic treatment and/or could make it easier to refer the patient to a professional that can deliver psychotherapy or to engage in collaborative care. Indeed, it has been shown in other circumstances that the actions of GPs could be influenced by the resources available in an immediate environment [69].

The use of treatment algorithms by all or most of the GPs for patients with depression is also a factor associated with adequate treatment at the clinic level. This is consistent with the results of Wells et al. [8] who showed that standardized treatment guidelines lead to better quality of care, but also improved clinical outcome, mental health– related functioning, and retention in employment of depressed patients. Use of treatment algorithms can also be viewed as a reflect of GP knowledge to treat depression. Previous studies reported that clinicians with greater knowledge about treatment of depression were more likely to care for patients with depression and to deliver high-quality depression care [70,71].

It has been demonstrated that payment mechanisms and financial incentives have significant effects on clinical decision making [72,73]. It is therefore not surprising that this barrier is associated with the quality of the treatment of depression in our study. Moreover, this is consistent with the findings of Dickinson et al. [33] who demonstrated that clinicians who provide more care for chronic medical problems may have a practice style, based on a model such as the chronic care model [74] and mostly associated with payment by capitation, that makes them more willing to provide greater depression treatment intensity to their patients.

Henke et al. [45] underscored that even highly trained and motivated clinicians will have difficulty providing guideline-concordant depression practices without support from their clinic. Our results support this notion, as we found both variables related to the clinicians knowledge (use of treatment algorithms) and related to the clinic's organization (psychotherapy on-site and mode of remuneration) associated with the quality of depression treatment.

### Limitations

The "Dialogue" Project provided high quality data on common mental disorders and service utilization. Nevertheless, results should be interpreted in light of the characteristics of the present study, which was based on self-reported cross sectional data.

Self-report data on the utilization of mental health services are subject to social desirability and recall bias [75] even though investigators reported acceptable concordance between self-report and administrative data [76,77]. In this study, we were unable to assess the reliability of the self-reported data, by a comparison with case notes for example. However, efforts were made to help participants answering accurately. For example, for the medication dosage, participants were asked to get and read their prescription or their pills box.

Temporality constitutes another limitation of cross-sectional studies. Participants that were diagnosed shortly before the administration of the survey may have just begun treatment and may not have had enough time to fulfill the consultation requirements. Likewise, services may have been received by participants before the onset of their depression.

Comorbidity with other mental health disorders is frequent. Some indicators may include care received for those comorbid conditions. Also, our definition of minimally adequate treatment did not consider the attitude of patients, such as non-compliance to or refusal of treatment; nor did it consider particular cases for which deviating from the guidelines may have been appropriate.

Due to the sampling strategy, our sample may not represent the entire population of adults consulting a GP in Quebec. The clinics response rate was less than 25%. Generalization of the results of this study is therefore limited.

Although our measure of minimally adequate treatment has not been directly linked to outcomes, it is concordant with guideline recommendations for high-quality depression treatment [19]. However, future research should focus on the validation of these quality indicators, for instance by studying their association with outcomes such as the reduction in depressive symptoms.

## Conclusions

This study enabled us to assess a large range of quality indicators covering many important components of depression treatment, and to examine the factors associated with treatment adequacy in primary care at the patient and the clinic levels. We found notable strengths in the care received by the patients. However, we also found important areas for quality improvement. As a large proportion of depressed people receive treatment only in primary care, the potential impact of targeting those indicators in this sector is all the more relevant.

This study also highlights an important public health problem: among patients reporting past-year MDE, only 1 out of 2 receive minimally adequate treatment. Our findings suggest that interventions are needed to increase the extent to which primary mental health care conforms to evidence-based recommendations. These interventions should target specific populations (i.e. the younger adults and the elderly), enhance accessibility to psychotherapy and to a regular family physician, and support primary care physicians in their clinical practice with patients suffering from depression in different ways such as developing knowledge to treat depression and adapting mode of remuneration.

## Competing interests

The authors declare that they have no competing interests.

## Authors' contributions

AD participated in the coordination of the study, performed the statistical analysis and drafted the manuscript. LF conceived the study, participated in its design and coordination and helped to draft the manuscript. LG participated in the statistical analysis and helped to draft the manuscript. PR participated in the design and coordination of the study, and helped to draft the manuscript. All authors read and approved the final manuscript.

## Acknowledgements

The Dialogue Project was funded by the Canadian Health Services Research Foundation (CHSRF), the Fonds de la recherche en santé du Québec (FRSQ), the Institut national de santé publique du Québec (INSPQ), the Groupe interuniversitaire de recherche sur les urgences (GIRU) and the Ministry of Health and Social Services of Quebec.

AD received PhD grants from the Fonds de Recherche en Santé du Québec (FRSQ), the Research in Addictions and Mental Health Policy & Services (RAMHPS), the Analyse et Évaluation des Interventions en Santé (AnEIS) and the Groupe de Recherche sur l'Équité d'Accès et l'Organisation des Services de Première Ligne (GREAS1).

LF holds an Applied Public Health Chair on population mental health from the Canadian Institutes of Health Research (CIHR), the Fonds de Recherche en Santé du Québec (FRSQ) and the Ministry of Health and Social Services of Quebec.

LG holds a CIHR/CRPO (Canadian Institutes of Health Research/Centre de recherche en prévention de l’obésité) Applied Public Health Chair on Neighborhoods, Lifestyle, and Healthy Body Weight.

PR holds a FRSQ Junior 1 new investigator award.

## Author details

^1^CRCHUM (Centre de recherche du Centre Hospitalier de l’Université de Montréal), Edouard-Asselin Pavilion, 264, René-Lévesque Blvd. East, Montreal, QC, Canada H2X 1P1. ^2^Université de Montréal, C.P. 6128, succursale Centre-ville, H3C 3 J7, Montreal, QC, Canada. ^3^Institut National de Santé Publique du Québec, 190 Crémazie Blvd. East, H2P 1E2, Montreal, QC, Canada. ^4^Université de Sherbrooke, 3001, 12e Avenue Nord, J1H 5 N4, Sherbrooke, QC, Canada.

## Pre-publication history

The pre-publication history for this paper can be accessed here:

http://www.biomedcentral.com/1471-244X/12/142/prepub
